# Transcriptomic Profiling the Effects of Airway Exposure of Zinc Oxide and Silver Nanoparticles in Mouse Lungs

**DOI:** 10.3390/ijms24065183

**Published:** 2023-03-08

**Authors:** Lan Zhao, Shuyuan Wang, Marit Ilves, Sanna Lehtonen, Leena Saikko, Hani El-Nezami, Harri Alenius, Piia Karisola

**Affiliations:** 1 Human Microbiome Research (HUMI), Medical Faculty, University of Helsinki, 00014 Helsinki, Finland; 2 School of Biological Sciences, University of Hong Kong, Hong Kong, China; 3 Department of Pathology, Medical Faculty, University of Helsinki, 00014 Helsinki, Finland; 4 Research Program for Clinical and Molecular Metabolism, Medical Faculty, University of Helsinki, 00014 Helsinki, Finland; 5 Institute of Environmental Medicine (IMM), Karolinska Institutet, 171 77 Stockholm, Sweden

**Keywords:** animal model, immune effects, lung, silver nanoparticles, transcriptomics, zinc oxide nanoparticles

## Abstract

Consumers and manufacturers are exposed to nanosized zinc oxide (nZnO) and silver particles (nAg) via airways, but their biological effects are still not fully elucidated. To understand the immune effects, we exposed mice to 2, 10, or 50 μg of nZnO or nAg by oropharyngeal aspiration and analyzed the global gene expression profiles and immunopathological changes in the lungs after 1, 7, or 28 days. Our results show that the kinetics of responses varied in the lungs. Exposure to nZnO resulted in the highest accumulation of F4/80- and CD3-positive cells, and the largest number of differentially expressed genes (DEGs) were identified after day 1, while exposure to nAg caused peak responses at day 7. Additionally, nZnO mainly activated the innate immune responses leading to acute inflammation, whereas the nAg activated both innate and adaptive immune pathways, with long-lasting effects. This kinetic-profiling study provides an important data source to understand the cellular and molecular processes underlying nZnO- and nAg-induced transcriptomic changes, which lead to the characterization of the corresponding biological and toxicological effects of nZnO and nAg in the lungs. These findings could improve science-based hazard and risk assessment and the development of safe applications of engineered nanomaterials (ENMs), e.g., in biomedical applications.

## 1. Introduction

The development of nanotechnology and ENMs, which have at least one dimension less than 100 nm, gives us great possibilities for enhanced applications in industry, medicine, and everyday products. Nanosized metal and metal oxide are used in the electronic industry for chips and sensors, biosensors, sorbent materials for environmental toxins, drugs and drug transport, and lubricants, which makes it easy for humans to be exposed to them [1,2]. Nanosized ZnO (nZnO) and Ag (nAg) particles are widely used, e.g., in medical catheters, healthcare and hygiene sprays, and textiles, because of their antibacterial properties [3,4,5,6,7]. ENM toxicity depends on the route of exposure (dermal, oral, inhalation, or injection) and exposure time [8]. Often, factory workers, in particular, can be exposed to nZnO and nAg through inhalation as a dry aerosol in the process of production, use, and recycling of ENM-containing products [9]. A worker in a mobile phone factory was reported to have remarkable serum Ag concentrations due to Ag-aerosol exposure in the workplace [10]. In addition, consumers will also have a risk of ENM inhalation while using antibacterial nAg-containing sprays, paints, or nAg-coated clothes [11]. Therefore, increased usage of nZnO and nAg raises concerns about their potential adverse effects on humans and potential toxicity in exposed target tissues, especially airways [12].

ENMs stimulate or suppress immune responses depending on their specific properties (surface chemistry, structure, agglomeration), and at present, a large number of studies have focused on the toxicity and impacts of nZnO and nAg in different lung models [13,14,15]. A study on human volunteers showed that two hours of inhalation exposure of 2.0 mg/m^3^ nZnO led to increased numbers of neutrophils, monocytes, and acute-phase proteins in the blood twenty-two hours after the exposure, and these effects lasted for three days after the exposure [16]. Occupationally relevant levels of 4.9 or 1.1 mg/m^3^ nZnO (51 nm or 48 nm) exposure to rats for 1, 7, and 30 days induced inflammatory and oxidative responses [17]. It also increased the quantities of the total cells, neutrophils, and total protein levels in bronchoalveolar lavage fluid (BALF) [17]. The nAgs were found in macrophages from BALF even 56 days after a single-dose aerosol of nAg (20 nm) to rats for 5.4 and 7.2 mg/m^3^ [18]. A 0.7 mg/m^3^ nAg (25 nm) inhalation exposure for 45 days accelerated lung cellular senescence and caused mild fibrosis [19]. Additionally, in another study, the number of neutrophils increased in BALF after 3.3 mg/m^3^ nAg (18–21 nm) inhalation exposure to mice for ten days [20].

Nanosized ZnO and especially Ag have features and potential for the development of novel antimicrobial agents, drug-delivery formulations, detection and diagnosis platforms, biomaterial and medical device coatings, tissue restoration and regeneration materials, and complex healthcare condition strategies. Given the impressive biomedical-related potential applications of nAg, efforts are taken to understand the intricate mechanisms of their biological interactions and possible toxic effects in vitro or in vivo. Still, a limited number of studies have focused on the inflammatory potential of a single exposure to nZnO and nAg at different doses and different exposure times in the airways over time. Furthermore, transcriptomic profiles on the airways induced by these nanoparticles are insufficiently studied, especially using in vivo setups. Therefore, we studied the inflammatory potential of exposure to nZnO and nAg at different doses (2, 10, or 50 μg) and observed biological effects after 1, 7, or 28 days in our mouse model of airway exposure. In addition to histological and cellular changes, we analyzed the transcriptome of mouse lungs to better understand changes in gene expression after exposure to nZnO and nAg. This kinetic in vivo study provides an important source to understand the cellular and molecular processes underlying nZnO- and nAg-induced changes in gene expression. Our study also characterizes the corresponding biological and toxicological effects of nZnO and nAg in the lungs, which enhances our knowledge of immune effects for further hazard assessment.

## 2. Results

### 2.1. Histological and Immunohistochemical Examination Showed an Accumulation of Immune Cells in Mouse Lungs after Exposure to nZnO and nAg

**Figure 1:**
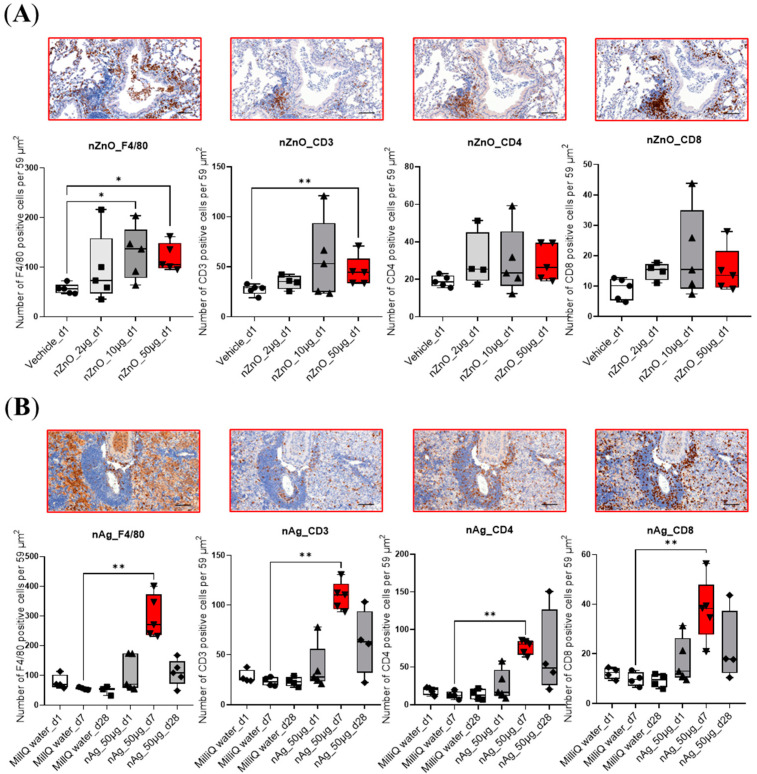
Immunohistochemical staining of mouse lungs exposed to nZnO and nAg for F4/80, CD3, CD4, and CD8. The number of the F4/80-, CD3-, CD4-, or CD8-positive cells per 59 μm^2^ are shown in (**A**) 1 day after exposure to 2, 10, or 50 µg of nZnO-, and (**B**) 1, 7, or 28 days after exposure to 50 µg of nAg. Immunohistochemical staining of one representative mouse lung is shown for nZnO 50 µg at day 1, and nAg 50 µg at day 7 (both bars indicated by red color). QuPath program was used for cell counting, and Mann–Whitney U-test was used for statistical analyses; * *p* < 0.05; ** *p* < 0.001 when compared to control groups. Scale bar: 100 µm.

Hematoxylin- and eosin-stained (H&E) lung sections were used to study the varying degrees of pulmonary inflammation induced by different exposures. The representative lung sections are shown in Figure S1. After exposure to 50 μg nZnO, the lungs showed the most severe inflammation compared to 2 μg or 10 μg exposure groups after 1 day (Figure S1A). However, after exposure to 50 μg nAg, the most severe inflammation occurred after 7 days rather than after 1 or 28 days (Figure S1B). The inflammation of mouse lungs was further studied by immunohistochemical staining, targeting CD3-, CD4- and CD8-positive T cells, as well as F4/80-positive cells, which represent an innate immunity component of the pulmonary inflammation mainly consisting of macrophages and some eosinophils (Figure 1). The number of F4/80 positive cells was increased in 50 μg and 10 μg nZnO exposure groups, while only 50 μg nZnO exposure showed a significant increase in the number of CD3-positive cells compared to the control group (Figure 1A). The numbers of F4/80-, CD3-, CD4-, and CD8-positive cells were increased significantly in the 50 μg nAg exposure group after 7 days when compared to the control group (Figure 1B). Collectively, these data indicate that the highest increase in cell numbers for all studied cell types was found after exposure to 50 μg of nZnO on day 1 and to 50 μg of nAg on day 7.

### 2.2. Gene Expression Varied Considerably between nZnO and nAg Exposure

**Figure 2:**
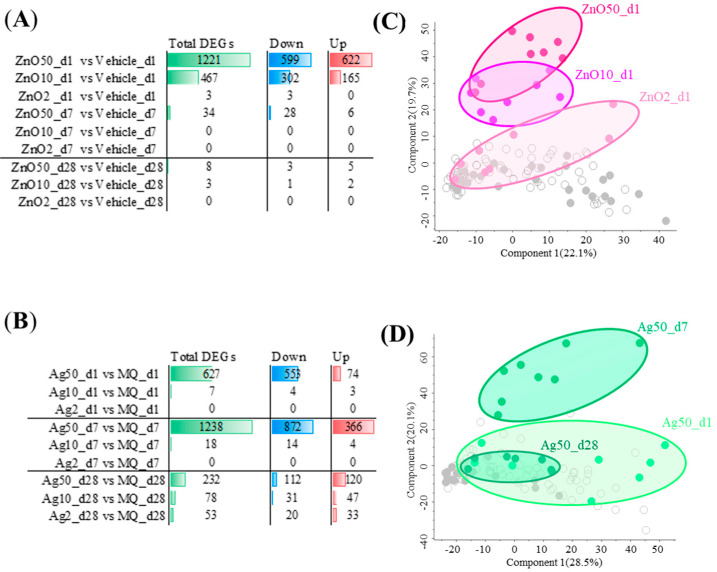
Exposure to nZnO and nAg causes varying numbers of differentially expressed genes (DEGs) in mouse lung tissue and induces changes in associated principal component analysis (PCA). The exposure to (**A**) nZnO or (**B**) nAg yielded a different number of total DEGs, which were either up- or down-regulated. When a fold change was ≥|1.25| and Benjamini-Hochberg adj. *p*-value was ≤0.05, DEGs were considered significantly different. The first two components are shown in (**C**) for nZnO and in (**D**) for nAg in PCAs. In (**C**), dark red filled circles represent 50 µg of nZnO, red 10 µg of nZnO, and light red 2 µg of nZnO 1 day after the exposure. In (**D**), dark green filled circles represent the exposure after 1 day, green represents the exposure after 7 days, and light red represents the exposure after 28 days to 50 µg of nAg.

Exposure to nZnO and nAg caused varying numbers of differentially expressed genes (DEGs) with Benjamini–Hochberg adj. *p*-value ≤ 0.05 and a fold change ≥ |1.25| between groups (Figure 2). The exposure to 50 μg nZnO yielded the highest number of DEGs, 1221, of which 599 were up-regulated, and 622 were down-regulated after a 1-day exposure. The numbers of DEGs of 10 μg or 2 μg nZnO exposures after 1 day were 467 and 3, respectively. Exposure to 50 μg nZnO yielded only 34 DEGs after 7 days, of which most (28/34) were up-regulated (Figure 2A). Only 8 (50 μg) or 3 (10 μg) DEGs were identified altogether after 28 days (Figure 2A). Interestingly, the exposure of 50 μg of nAg showed the highest numbers of DEGs 7 days after the nAg exposure, a total of 1238 DEGs, of which 872 DEGs were up-regulated and 366 were down-regulated (Figure 2B). The two-dimensional clustering of nZnO or nAg exposure is demonstrated in PCAs (Figure 2C,D). The 50 μg or 10 μg exposures of nZnO were distinguished from other groups in the PCA after 1 day, of which the 50 μg nZnO group exhibited the most distinct gene expression pattern (Figure 2C). In the PCA of nAg, the exposure group representing responses after 7 days of exposure was the most prominent (Figure 2D).

Since lung inflammation and DEGs were seen only 1 day after the nZnO exposure but not 7 or 28 days after the exposure, a further detailed analysis was possible only with 1-day groups. Similarly, since lung inflammation and DEGs were seen only after exposure with the 50 μg nAg but not with the 10 and 2 μg nAg, further bioinformatic analysis was possible only with a 50 μg dose. Thus, further analysis in the rest of our studies will be shown only for 1-day groups after the nZnO exposure with all doses and only for 50 μg-dose groups 1, 7, and 28 days after the nAg exposure.

### 2.3. The nZnO-Specific DEGs Are Enriched in Innate Immunity Pathways

**Figure 3:**
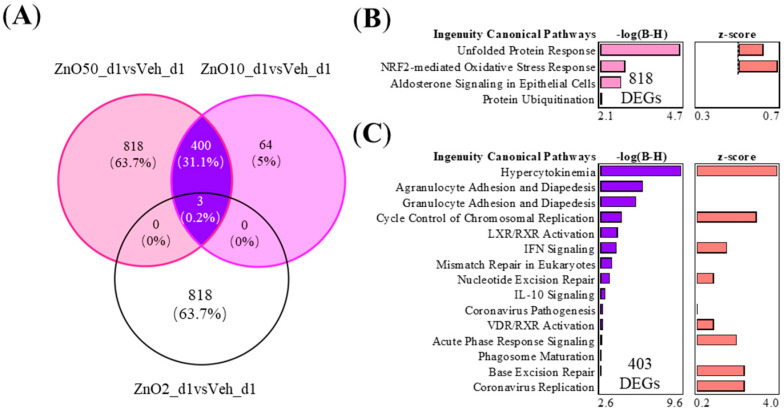
The nZnO-treated mouse lung tissues have specific or shared gene expressions with varying functions depending on the particle concentration 1 day after the exposure. (**A**) The exposure to nZnO yielded 403 shared and 818 or 64 specific DEGs in the VENN diagram for nZnO 50 µg or nZnO 10 µg, respectively. The different canonical pathways (IPA) are shown in (**B**) for 818 DEGs, which are exclusively expressed after exposure to 50 µg of nZnO, and in (**C**) for 403 DEGs after exposure to 10 or 50 µg of nZnO. The negative logarithm of the *p*-value calculated by the right-tailed Benjamini-Hochberg test is equal to increased significance. The predicted pathway activation (red bars) or inhibition are represented in the z-score bar chart.

Shared and unique DEGs between the 2 μg, 10 μg, and 50 μg nZnO exposure groups 1 day after the exposure are shown in Figure 3A. The 50 μg nZnO exposure group showed 818 unique DEGs, which were enriched in Ingenuity Pathway Analysis (IPA) to cellular stress responses such as endoplasmic reticulum (ER) stress and oxidative stress and ubiquitin modifications (Figure 3B). The 50 μg nZnO exposure group and the 10 μg nZnO exposure group shared 403 DEGs that were mainly predicted to be enriched to innate immunity, including cytokine storm and the migration of different types of leukocytes (Figure 3C). These shared 403 DEGs were predicted to activate upstream regulators, including IFN-γ, TNF, and IL-4 (Figure S2A). The 10 μg nZnO exposure group had 64 unique DEGs, but no pathway enrichment was seen in IPA.

### 2.4. The Specific 1142 DEGs of 50 μg nAg after 7 Days of Exposure Are Related to Adaptive Immunity

**Figure 4:**
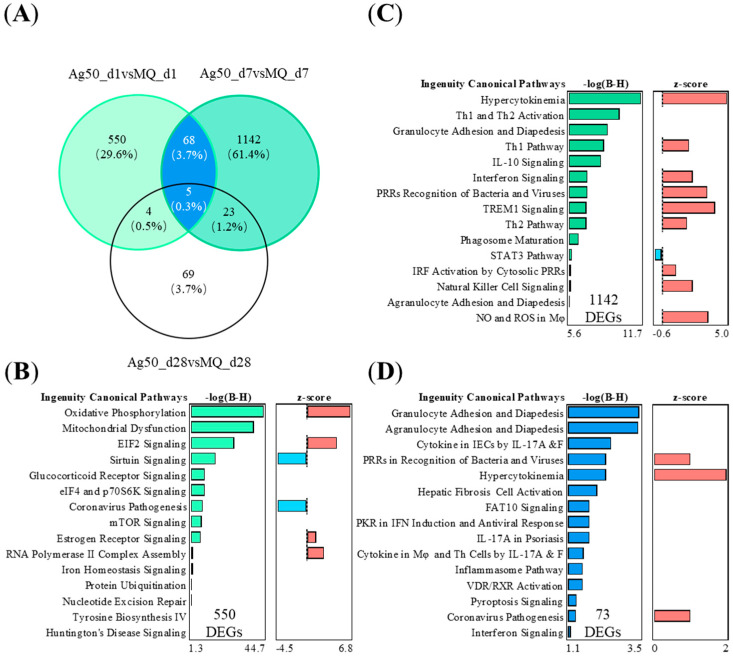
The nAg-treated mouse lung tissues have specific or shared gene expressions with varying functions depending on the time after the 50 µg of nAg exposure. (**A**) The exposure to nAg yielded 73 shared and 550 or 1142 specific DEGs in the VENN diagram for nAg 1 or 7 days after the exposure, respectively. NAg 28 days after the exposure yielded 101 DEGs. Five DEGs were shared between all the groups. The different canonical pathways (IPA) are shown in (**B**) for 550 DEGs exclusively expressed 7 days after the exposure, in (**C**) for 1142 DEGs exclusively 1 day after the exposure, and in (**D**) for the shared 73 DEGs 1 and 7 days after the exposure. The negative logarithm of the *p*-value calculated by the right-tailed Benjamini-Hochberg test is equal to increased significance. The predicted pathway activation (red) or inhibition (blue) are represented in the z-score bar chart.

To study the nAg-driven effects in mouse lungs at different time points, we studied the shared and unique DEGs between 50 μg nAg exposure groups after 1, 7, or 28 days (Figure 4A). The nAg group after 1 day showed 550 specific DEGs, and the nAg exposure group after 7 days showed 1142 specific DEGs, respectively. In the 50 μg nAg exposure group, after 1 day, the 550 DEGs were predicted to activate the enriched pathways, including oxidation-reduction reactions and dysfunction of mitochondria and protein translation initiation (Figure 4B). After 7 days, the specific 1142 DEGs enriched into the pathways playing a role in activating innate immunity, such as cytokine storm, and adaptive immunity, such as the activation of Th1 and Th2 (Figure 4C). The nAg exposure groups after 1 and 7 days shared 73 common DEGs, which enriched to migration of different types of leukocytes and cytokine regulation by Th17 (Figure 4D). These shared 73 DEGs of nAg exposure groups activated upstream regulators IL-1β, IL-10, and IL-6 (Figure S2B).

### 2.5. Common and Specific Gene Expression Modules after Exposure to nZnO and nAg

**Figure 5:**
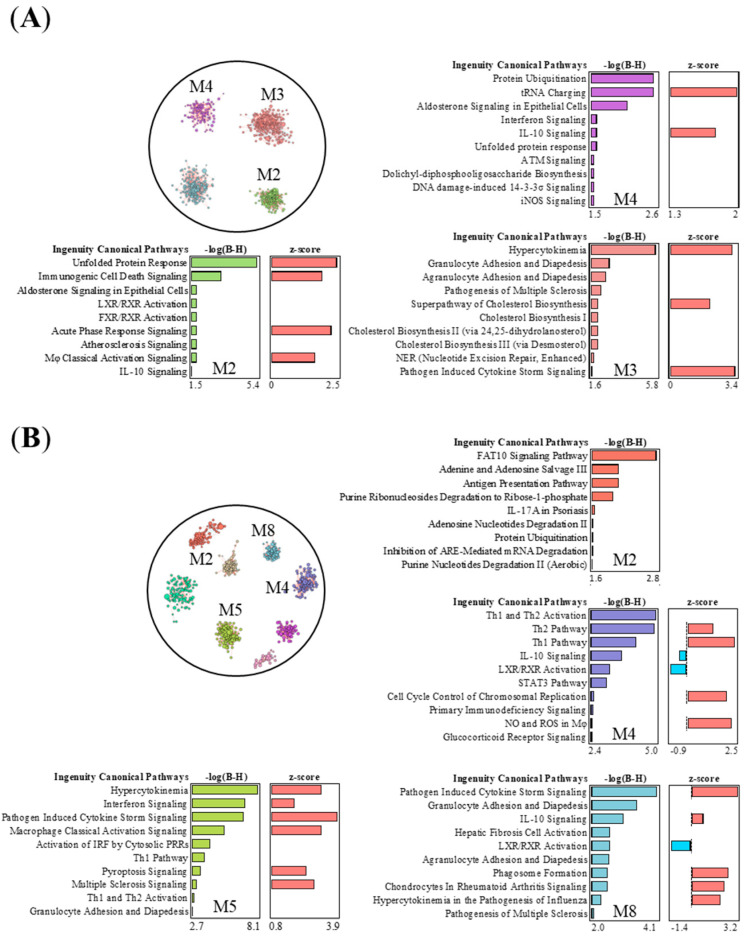
The nZnO- and nAg-treated mouse lung tissues have specific or shared response interaction modules and corresponding canonical pathways. Pathways of the negative logarithmic of the *p*-value calculated by the right-tailed Benjamini-Hochberg (B-H) larger than 1.5 are shown, and module color is matched with associated -log(B-H)-bars. (**A**) There are four modules in the top 700 DEGs of 50 μg nZnO exposure group after 1 day, and three modules yielded the canonical pathways. (**B**) There are eight modules in the top 700 DEGs of 50 μg nAg exposure group after 7 days, and four modules yielded the canonical pathways. The top 700 DEGs were selected for nZnO and nAg groups for INfORM to analyze network response modules, and then the canonical pathways analysis was completed by IPA.

To study whether the most prominent effects triggered by nZnO and nAg show similarities or differences in the function of expression modules, we compared the top 700 DEGs of the 50 μg nZnO exposure group after 1 day and the 50 μg nAg exposure group after 7 days and performed gene network analysis, response module detection, and prediction of module function by canonical pathway analysis (IPA) (Figure 5). Four modules were detected in the group of 50 μg nZnO after 1 day (Figure 5A). Module 2 was related to endoplasmic reticulum stress and inflammatory responses such as immunogenic cell death and acute phase response (Figure 5A). Module 3 was enriched to cytokine storm and the migration of different types of leukocytes, while module 4 was enriched to ubiquitin modifications, amino acid activation, and cytokine signaling (Figure 5A). Exposure to 50 μg nAg after 7 days yielded eight modules (Figure 5B). Module 2 was related to ubiquitin modifications, cell metabolism, and antigen presentation, while module 4 was enriched to activation of Th1 and Th2 and the signaling of IL-10 (Figure 5B). Module 5 was related to cytokines, “interferon signaling”, and cytokine storm signaling induced by pathogens, classical activation of macrophages, and the activation of the innate immune response (Figure 5B). Additionally, module 8 was enriched to the signaling of cytokine storm induced by pathogens (Figure 5B).

## 3. Discussion

Due to the increased use and production of nanomaterial-derived consumer products, airway exposure to ENM particles is increased [21]. Airway exposure is a non-negligible route of ENM exposure; people may be exposed to ENM during the process of product manufacturing and daily life [22,23,24]. A previous review has summarized the toxicity mechanism of nanoparticles, including oxidative stress, inflammatory response, and DNA damage in systemic (animal) and cellular studies [25]. We and others have also found that ENMs may induce versatile immune responses in the process of antigen recognition and uptake, as well as in the developed innate and adaptive immune responses in the gut [26,27] and skin [28,29]. In the present study, we explored whether similar biological effects and mechanisms are developed after exposure to nZnO and nAg in the airways. We performed a series of oropharyngeal aspiration exposures of different doses to mice and analyzed the immunohistochemical and transcriptional changes in the lungs after different time periods.

Dose-dependent accumulation of inflammatory cells, especially F4/80-positive cells and CD3-positive T cells near large airways, was especially prominent 1 day after exposure to nZnO. In the nAg exposure groups, the most intense lung inflammation was seen on the 7th day after the exposure. There was also large infiltration of F4/80-positive cells and both CD4- and CD8-positive T cells in the lower parts of the lungs. After 28 days of exposure, some infiltrating cells could still be seen, suggesting that the effects of nAg may persist even one month after the exposure. In line with this, others have also found pulmonary inflammatory responses accompanied by an increase in macrophages, neutrophils, and lymphocytes in the mouse BALF after nZnO or nAg airway exposure [30,31,32]. The transcriptional changes in mouse lungs supported the differences seen in the histological examination. The largest number of gene expression changes occurred after nZnO (1221 DEGs) or nAg (1238 DEGs) at the 50 μg exposure dose, but for nZnO at day 1 and for nAg at day 7. These results suggest that the kinetics and inflammatory mechanisms induced by nZnO and nAg are somewhat different, although they are both metal oxide nanoparticles and appear to be the same in size.

Closer analysis showed that nZnO induced the strongest inflammatory response of all the studied exposure doses 1 day after aspiration. We found that the shared 403 DEGs between 10 and 50 μg exposure doses after nZnO exposure on day 1 stimulated chemotaxis and interferon signaling. This suggests that nZnO exposure stimulates the rapid release of proinflammatory cytokines and acute phase proteins from macrophages and epithelial cells, resulting in the development of strong acute airway inflammation [33]. This was further supported by the increased accumulation of F4/80 cells (mainly macrophages) in mouse lungs. Together, these events could initiate a sustained or progressive inflammatory process leading to lung injury [34]. A study of 3.3 ± 0.6 mg/m^3^ nZnO (10 nm) inhalation exposure to mice for 13 weeks also caused an increase in macrophages in BALF and a moderate increase in IL-12 [35]. In line with our findings, another study of single inhalation exposure to 300 μg of nZnO (20 nm) in rats caused acute lung injury with granulocyte accumulation and also showed significantly increased proinflammatory IL-6 and TNF production to the BALF of rat lungs after intratracheal instillation of nZnO at 1 day [36]. Exposure of mice to 2, 6, and 18 μg of nZnO (12 ± 3 nm) by a single intratracheal instillation after 3 days caused a very strong inflammatory response and increase in neutrophils in BALF [37]. Additionally, the 818 DEGs (specific only for the 50 μg of nZnO at day 1) revealed significant enrichment in stress pathways, such as ER stress activation and NRF2-mediated oxidative stress response. Oxidative stress is caused by the excessive formation of reactive oxygen species (ROS) and impairment of defensive antioxidant systems, which disrupt mitochondrial function and contribute to cellular damage and death [38]. It has been reported that metal oxide nanoparticles such as nZnO may directly produce ROS and lead to oxidative stress [39]. Therefore, it seems that nZnO airway exposure may induce innate immune responses leading to acute inflammation and the development of oxidative stress responses.

Compared to nZnO exposure, nAg exposure induced broader immune responses by activating both innate and adaptive branches of immunity. The shared 73 DEGs (in 50 μg nAg exposure groups after 1 or 7 days) enriched the innate immune responses such as the adhesion and diapedesis of immune cells, hypercytokinemia, and interferon signaling. In our previous studies, 24 h of 100 µg/mL nAg (20 ± 3 nm) exposure to THP-1 cells induced the expression of genes related to virus recognition and type I interferon responses [40]. In addition, the activation of the IL-17 pathway was observed in our current studies. Nanosized Ag may regulate macrophages and helper T (Th) cells via IL-17 to enhance immune responses, which activate neutrophils and induce inflammation [41]. Seiffert et al. also found that the inhalation of 13–16 nm 600–800 μg/mm^3^ (equal to 8–28 μg lung burden) nAg induced an acute pulmonary neutrophilic inflammation that was associated with the production of proinflammatory and pro-neutrophilic (IL-17A) cytokines in rats after 1 day [42]. In contrast, 550 DEGs (in 50 μg nAg exposure group after 1 day) of nAg exposure demonstrated the enrichment of pathways related to oxidative phosphorylation and mitochondrial dysfunction, which are related to the energy production system and metabolic functions. In addition to early-induced innate immunity responses, Th1 and Th2 activation pathways related to adaptive immunity were strongly activated, especially in the nAg exposure group after 7 days (1142 DEGs only in the 50 μg nAg exposure group after 7 days). Th1-differentiating transcription factor STAT1 and cytokine IFN-γ, as well as Th2-prone IL-4, were also predicted to be activated by these nAg-specific DEGs. A pathway analysis of 670 ± 49 ng/cm^2^ aerosol nAg (14.1 ± 2.3 nm) inhalation exposure to rats after 1 or 7 days enriched the same pathways related to granulocyte and agranulocyte adhesion and diapedesis, IL-10 signaling and hypercytokinemia [43]. Another study found that 50 μg (20 nm) citrate-coated nAg exposure to mice elevated *IL-4* and *IL-10* gene expression after 7 days [44]. Additionally, a study using an in vitro alveolar model composed of an alveolar type-II cell line (A549), differentiated macrophage-like cells (THP-1), mast cells (HMC-1), and endothelial cells (EA.Hy926) found that 0.5 and 5 μg/cm^2^ PVP-coated nAg (50 nm) increased the secretion of IL-4, IL-6, and IL-10 after 6 h and 24 h [45]. In conclusion, nAg activates both innate and adaptive immune pathways, and the developed responses last longer (up to 28 days) than after nZnO exposure (only 1 day).

To explore whether the strongest effects of nZnO (50 μg nZnO group after 1 day) and nAg (50 μg nAg group after 7 days) have some common or distinct mechanisms of functions, we focused on the interaction of gene response modules of the 50 μg nZnO group after 1 day of exposure and the 50 μg nAg group at day 7, which yielded the most transcriptional changes. We found some similarities in innate immune pathways induced by nZnO or nAg exposure, suggesting that many of the observed effects share common mechanisms. Both nZnO (module 3) and nAg (modules 5 and 8) activated the chemotaxis of granulocytes and agranulocytes and cytokine production pathway, which are related to innate immune responses, including the recognition of bacteria and viruses (PRR), macrophage activation, and NK cell signaling. Interestingly, the activation of interferon signaling, a hallmark of the viral response [46], was also found after exposure to nZnO and nAg. In addition, nZnO exposure (in modules 2 and 4) activated acute phase responses and cell-death-related pathways. In contrast, nAg exposure significantly modulated the adaptive immune responses, especially Th1 and Th2 activation pathways. Similarly, silver nanorods were also shown to induce T cells to secrete more Th1 and Th2 cytokines [47]. Although the exact mechanisms are not known, it might be that nAg particles enhance antigen presentation, which leads to the maturation of naïve T cells and possibly also B cells. It can be speculated that the observed inflammatory effects of nZnO and nAg could be at least partly due to the release of ions after exposure [48,49]. Zinc serves as an important cofactor in the processes of protein synthesis in normal cells. The possible mode of toxicity for nAg might be the lysosomal degradation of internalized nAg and the released Ag iron interaction with biological systems [50]. Altogether, the studied differences between nZnO and nAg effects may be due to their physicochemical properties, especially their ion dissolution, which determines the timing and extent of cellular uptake, which have been suggested to have a significant impact on the activation mechanisms of immune responses by nZnO and nAg in the lung environment [51,52,53].

## 4. Materials and Methods

### 4.1. Nanoparticles

The nanosized ZnO was purchased from nanostructured & Amorphous Materials, Inc. (Garland, TX, USA), and the nanosized Ag was purchased from NanoComposix, Inc. (San Diego, CA, USA). The properties of nZnO and nAg are shown in Table 1, and their characterization has been done in our previous study [40].

The n-ZnO particles were weighed into autoclaved glass tubes, and the dilutions of the working suspension were prepared into a sterile DPBS in a concentration of 2 μg, 10 μg, and 50 μg. The n-Ag particles were dispersed in MilliQ-water, and the dilutions of the working suspension were prepared into DPBS in a concentration of 2 μg, 10 μg, and 50 μg.

### 4.2. Animal Procedures

Female C57BL/6JOlaHsd mice (7 weeks old) were purchased from Envigo Rms, Inc. (Indiana, USA) and quarantined for 7 days and housed in stainless steel cages with aspen chip bedding in groups of 4–6 mice for control groups and 8 for experimental groups. The housing conditions were carefully controlled with temperature (20–21 °C) and humidity (40–45%) and 12 h dark/light cycles. Mice received food and water ad libitum.

All animal experiments were performed by Finnish legislation by certified researchers. The experiments were done in agreement with the European Convention for the Protection of Vertebrate Animals Used for Experimental and Other Scientific Purposes (Strasbourg 18 March 1986, adopted in Finland 31 May 1990). All experiments were approved by the State Provincial Office of Southern Finland.

### 4.3. Oropharyngeal Aspiration Exposures

Mice were exposed by a single oropharyngeal aspiration under isoflurane anesthesia to 2, 10, or 50 μg of ENM (nZno or nAg) in 50 μL, which is equal to the doses in 0.1, 0.5, or 2.5 mg/kg body weight/day to stimulate human (60 kg) exposure dose as 10, 50, or 250 μg/mg [54]. Control mice for nZnO exposure groups received 50 μL of DPBS as the vehicle group, and control mice for nAg exposure groups received 50 μL MilliQ-water as the vehicle group. As nAg was pre-dissolved in the sterile water, we had to use it as a vehicle, whereas the nZnO dispersion was done in our laboratory from the nZnO powder, and DPBS could be used in both cases. The exposure method and doses were selected based on our previous studies [54,55]. We have previously demonstrated that oropharyngeal aspiration of ENM generates highly comparable results with airway inhalation exposure in terms of airway inflammation and global lung transcriptome [54], which justifies the use of this exposure method. Then, 1, 7, or 28 days after the exposure, mice were killed by an overdose of isoflurane, and lung samples were collected. The bottom half of the left lungs were stabilized in RNALater solution (Thermo Fisher Scientific, Inc., Waltham, MA, USA) at +4 °C for 24 h and stored at −80 ℃ until total-RNA was extracted. The upper part of the left lung was fixed in formalin for 24 h (to be used for histology and immunohistochemistry).

### 4.4. Histological and Immunohistochemical Stainings of Mouse Lungs

The formalin-fixed lungs were washed in 70% ethanol and subjected to standard dehydration processing to prepare them for mounting in paraffin wax blocks, and 3.5 µm sections were prepared for histological and immunohistochemical staining. After deparaffinization, the sections were stained with hematoxylin and eosin using a standard protocol. For immunohistochemistry, after deparaffinization and antigen retrieval with EnVision™ FLEX Target Retrieval Solution High pH (GV804; Dako, Santa Clara, CA, USA), the sections were stained with F4/80 Rabbit mAb (70076S) CD3ε Rabbit mAb (78588S), CD4 Rabbit mAb (25229S) and CD8α Rabbit (98941S), all from Cell Signaling Technology, Inc., Danvers, MA, USA. Detection was carried out using BrightVision 1-step detection system anti-rabbit HRP (VWRKDPVR55HRP, ImmunoLogic, Duiven, The Netherlands) and 3,3′-diaminobenzidine (Dako). Slides were counterstained with Mayer’s Hematoxylin (Dako).

### 4.5. Cell Counting

All staining images were generated using 3DHISTECH Pannoramic 250 FLASH II (3DHISTECH, Budapest, Hungary) digital slide scanner at Genome Biology Unit supported by HiLIFE and the Faculty of Medicine, University of Helsinki, and Biocenter Finland. The positive cell counting was completed by the QuPath v0.3.2 (QUB, Belfast, UK) program. Ten annotated lung areas were selected (four from lung surfaces, three near the large airway, two near the middle airway, and one near the small airway), and the positive cell detection function was used to identify, count, and analyze different cells.

### 4.6. RNA Extraction

Lung tissue samples were thawed and moved to LysingMatrix D tubes (MP Biomedicals, Illkirch, France) containing 1 mL of Trisure and homogenized by FastPrep FP120 homogenizer (Savant Bio 101, Thermo Fisher Scientific, Inc., Waltham, MA, USA). Then, chloroform was used for RNA phase separation, isopropyl alcohol was used for precipitation, 75% ethanol was used for washing, and DEPC-treated water was used for re-dissolving. RNA clean-up was used by NucleoSpin RNA Clean-up Machinery-Nagel GmbH & Co, Duran, Germany). The quantity and quality of the isolated total RNA were measured by NanoDrop spectrophotometer (ND-1000, Thermo Fisher Scientific Inc., Wilmington, NC, USA) and Agilent TapeStation (4200, Agilent Technologies, Inc., Santa Clara, CA, USA), respectively. Samples with RNA integrity number (RIN) >8 were used for further analysis.

### 4.7. RNA Sequencing

The Nextera XT DNA sample prep kit (Illumina, Inc., San Diego, CA, USA) was used to prepare the library according to the manufacturer’s instruction, and the 3′ end-amplified fragments were sequenced on the Illumina NextSeq 500 platform. Filtered and trimmed sequence reads less than 20 nt were trimmed with the help of the tool Trimmomatic (parameters: LEADING:3, TRAILING:3, SLIDING WINDOW:4:15, and MINLEN:36). PolyA tails of length six or greater were removed in the Drop-seq tools (https://github.com/broadinstitute/Drop-seq, accessed on 22 February 2021). Then the obtained sequences were mapped to the GRCm38.p6 whole genome by using STAR (v2.6.0a, MIT, Cambridge, MA, USA) with the default settings for gene annotation. FeatureCounts software (v1.6.4, UniMelb, Melbourne, VIC, Australia) was used to calculate raw read counts.

The differential gene expression was analyzed in Chipster (CSC-IT Science Center, Espoo, Finland). A TMM normalization was performed, negative binomial generalized linear models and Wald test were fitted, and Cook’s distance was used to delete outliers in DESeq2. The significance threshold of differentially expressed genes (DEGs) was set at fold change ≥ |1.25|, and Benjamini-Hochberg posthoc correction was adjusted as *p* ≤ 0.05.

### 4.8. Data Analysis

Principal component analysis (PCA) and heatmap clustering were conducted by Perseus (v2.0.3.1, Max-Planck-Institute of Biochemistry, Munich, Germany). The physiological implications of the DEGs identified for each exposed/unexposed contrast were predicted via canonical pathways, upstream analyses, or disease/function enrichment analyses using the Ingenuity Pathway Analysis (QIAGEN Inc., Hiden, Germany) pathway analysis tool. Only DEGs with a fold change ≥|1.25| and Benjamini-Hochberg adj. *p*-value ≤ 0.05 were used for pathway enrichment analyses. The module analysis of DEGs in different groups was conducted by INfORM (Inference of NetwOrk Response Modules, Tampere, Finland). Analysis and mapping of the difference in the number of positive cells in IHC sections were conducted by Prisma GraphPad Software (v8.0, Inc., San Diego, CA, USA).

## 5. Conclusions

We performed in-depth transcriptomic profiling of the effects of two metal nanoparticles (nZnO and nAg) at different doses (2, 10, or 50 μg) and different follow-up times (1, 7, and 28 days) using a mouse model of airway exposure. Transcriptomic profiles in the airways revealed that the exposure to nAg induced a wider immune response, especially at day 7, activating both innate and adaptive branches, than that of nZnO, which elicited innate immunity-dominated acute airway inflammation that peaked at day 1. Our analyses deepen the understanding of the predicted immunomodulating mechanisms of ENMs exposure and will benefit the development of safer applications of ENMs in various industry sectors, including healthcare, biomedicine, and consumer products.

## Figures and Tables

**Table 1:** Characteristic of nanosized ZnO and nanosized Ag.

	Type	Size	Form	SSA	Purity	Coating	Dissolution
n-ZnO	Nano	20 nm	Particle	50 m^2^/g	99.5%	-	Not provided
n-Ag	Nano	20 ± 3 nm	Dispersion in H_2_O	27.4 m^2^/g	99.99%	Polyvinylpyrrolidone (PVP)	1 ppb–2 ppm/H_2_O

## Data Availability

The data presented in this study are available on request from the corresponding author.

## Supplementary Materials

The supporting information can be downloaded at: https://www.mdpi.com/article/10.3390/ijms24065183/s1.
