# Supplementary figures and images for: Transcriptomic Profiling the Effects of Airway Exposure of Zinc Oxide and Silver Nanoparticles in Mouse Lungs
Source: Int J Mol Sci. 2023 Mar 8;24(6):5183. doi: 10.3390/ijms24065183 (PMC10049322; doi:10.3390/ijms24065183)

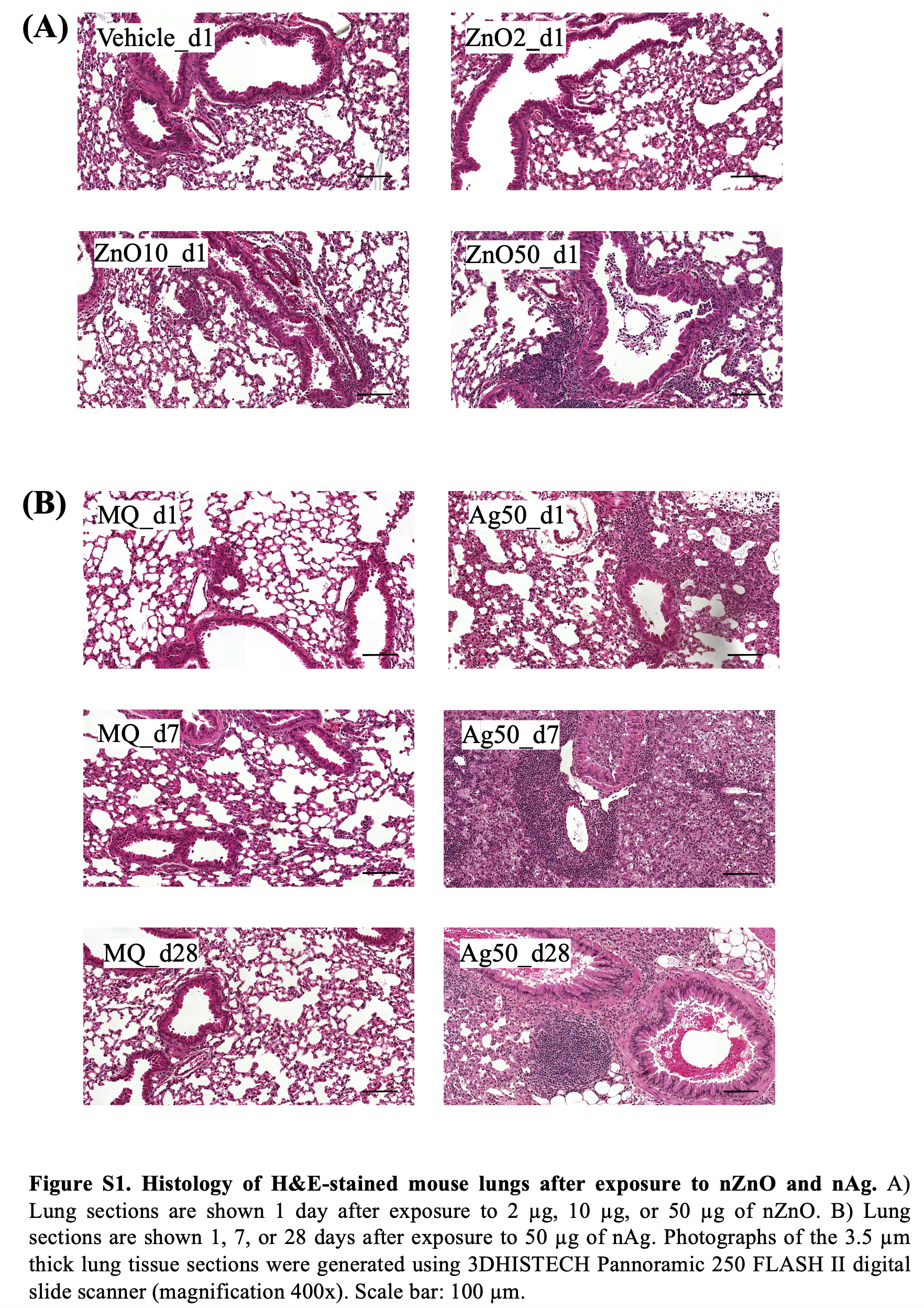

Supplement: Supplementary file 1 [file ijms-24-05183-s001.zip › Figure_S1.tiff]

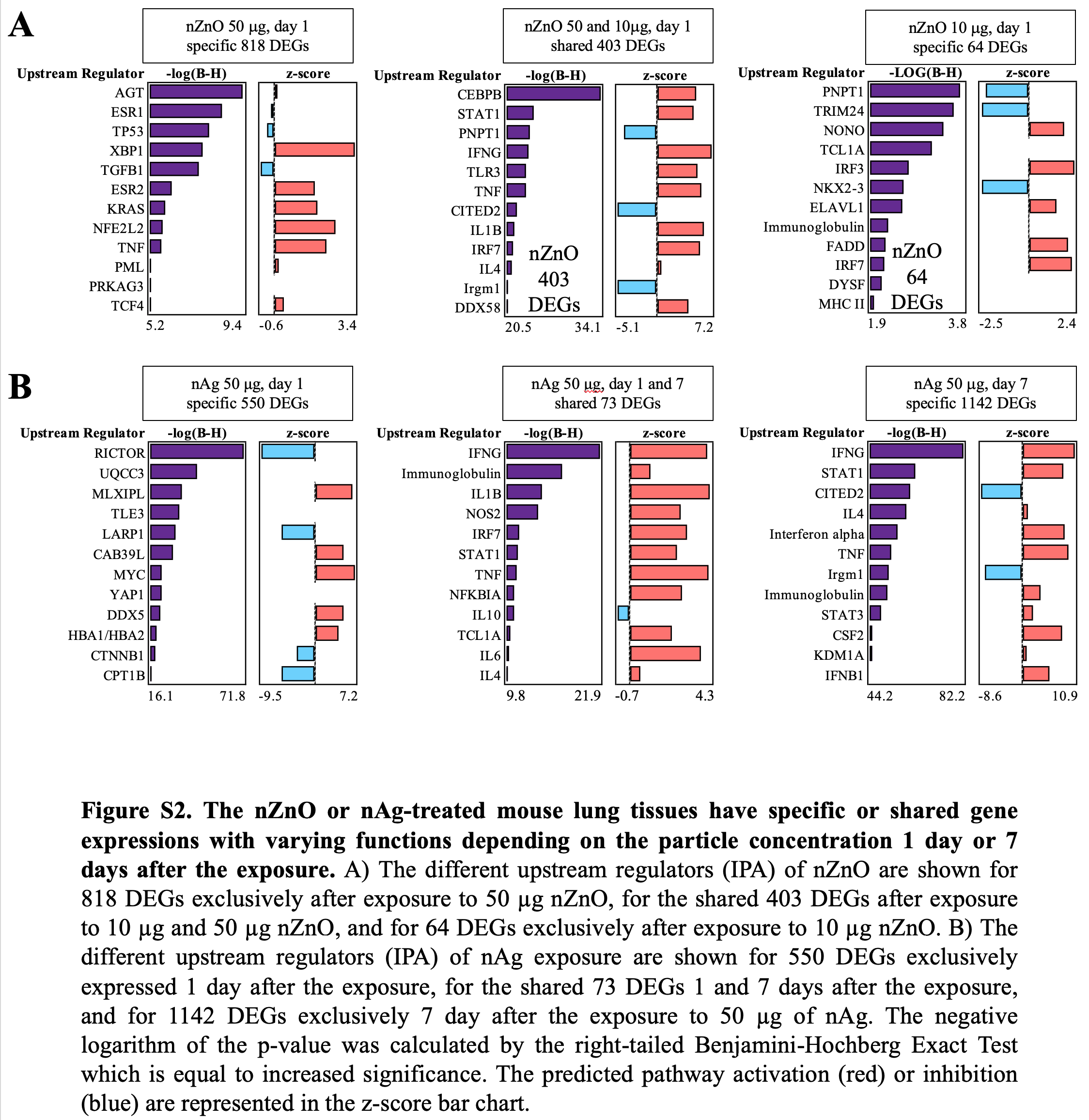

Supplement: Supplementary file 1 [file ijms-24-05183-s001.zip › Figure_S2.tiff]
